# Proceedings of the Stillbirth Summit 2023: Bridging the gap between research and practice

**DOI:** 10.1186/s12919-023-00274-x

**Published:** 2023-09-12

## O1: My quest to end preventable stillbirth in the US and how it has changed me

### Heather Florescue

#### Department of OB/GYN, University of Rochester, Rochester, New York, USA

*BMC Proceedings 2023,***17(14):**O1

**Introduction**

There is a fatalist view that stillbirth can't be prevented. In June of 2019, I attended the Star Legacy Foundation Stillbirth Summit and learned that this view is in correct. I learned that the United Kingdom and Australia have protocols that have succeeded in reducing their stillbirth rate. I set off to try to make these protocols a standard of care at my private practice and in my community. This four-year journey has not been easy because changing the status quo never is. I will discuss the model I used to try to change my community and give a framework for attendees on how to bring change to their communities from what they learned at the 2023 Stillbirth Summit.

**Methods**

Discuss the status quo, which is the existing state of affairs, especially regarding social and political issues. Also address the methods from the book *Switch* by Chip and Dan Heath that can be used to make a change to the status quo. To do this you need to motivate the elephant, direct the rider and shape this path.

**Conclusion**

Changing the Status Quo is hard but it is a marathon not a race. There will be losses and wins, but change is possible and worth it.

## O2: Reducing the risk of placenta-mediated stillbirths: A Placenta Clinic approach

### John C. Kingdom

#### Department of Obstetrics & Gynaecology, Mount Sinai Hospital, University of Toronto, Toronto, Canada

*BMC Proceedings 2023,***17(14):**O2

Stillbirth associated with underlying pathological disorders of the placenta represent the largest category of potentially-avoidable stillbirth. The prevalence of many placental diseases is steadily increasing in contemporary Obstetrics, and in tandem we have developed tools that may be effective to diagnose them and therefore deliver improved outcomes. However, despite the wide availability and increased use of ultrasound, current trends show only minimal improvements to the approximately 1/300 risk of losing a potentially-viable normally-formed fetus. The most common placental cause of stillbirth is maternal-vascular malperfusion (MVM) disease, characterized by a constellation of visible and microscopic features whose first trimester origins begin with failure to develop adequate uteroplacental blood flow. Multi-modal methods can now identify at-risk pregnancies across all three trimesters, and interventions such as low-dose aspirin prophylaxis in the first trimester, and targeted fetal monitoring based on screening in all trimesters has the potential to reduce the rate of avoidable stillbirth via the more effective diagnosis of fetal growth restriction. Other placental diseases present much greater challenges to clinicians. Two notable examples are Fetal Vascular Malperfusion (FVM) and Villitis of Unknown Etiology (VUE). Unlike, MVM, neither of these conditions has any maternal attributes. Both are associated with normal uterine artery Doppler waveforms and placenta growth factor (PlGF) levels and therefore do not cause hypertension, which is a useful clinical alert to provide fetal assessment by ultrasound. Impacting these underlying diseases is therefore only currently made possible via universal ultrasound screening in the 3rd trimester. Rarer diseases, notably chronic histiocytic inter-villositis (CHI) and massive perivillous fibrin deposition (MVPD) or maternal floor infarction of the placenta, begin early in pregnancy and are often initially manifested by multiply-abnormal enhanced first trimester screening (eFTS) for trisomy 21, a screening test that is at risk of disappearing in favor of the more effective NIPT. Ultrasound may in addition be useful in establishing the prenatal diagnosis of a range of additional placental diseases, for which the impact may be profound (as for vasa previa) or potentially life-saving for the mother (placenta previa-percreta). Collaboration between regional placenta-focused programs in Maternal- Fetal Medicine centers has the capacity to advance care beyond recommendations in existing clinical practice guidelines. In the next 5 years, it is within our grasp to make substantive gains in the prevention of placenta-mediated stillbirth due to MVM disease with existing tools, and to network in order to prioritize some common research goals across the full spectrum of diseases that mediate stillbirth.

## O3: Generating diagnostics that flag babies in peril from poor placental function

### Stephen Tong

#### Department of Obstetrics, University of Melbourne, Co-Director, Mercy Perinatal, Mercy Hospital for Women, Melbourne, Australia

*BMC Proceedings 2023,***17(14):**O3

We have a large research program focused on developing a blood test to prevent stillbirth. Our vision is to find circulating molecules in the mum's blood that flags pregnancies where placental function is waning. This will allow us to identify pregnancies at heightened risk of stillbirth. These vulnerable pregnancies could closely monitored, and the baby birthed before a stillbirth happens.

In this presentation, I will provide an overview in our team's multi-pronged efforts to develop a stillbirth prevention test. I will provide an update on our lead molecule, SPINT1 (Kaitu'u-Lino et al, Nature Communications 2020), including new data showing low SPINT1 levels in the mum's blood is not merely linked to small fetal size (a rough indicator of poor placental health), but also predicts severe neonatal complications after birth (more clinically relevant indicator of poor placental health).

I will overview the exciting breadth of science we are deploying to develop a stillbirth blood test. New generation technologies to hunt for more markers; cutting edge engineering to develop a 'point of care test', and artificial intelligence to enhance the diagnostic performance of our candidate biomarkers.

Finally, I will cover the challenges faced by any team working to develop a stillbirth prevention test – deciding the diagnostic thresholds to aim for, the haziness in defining 'placental insufficiency', and the level of evidence that may be needed to convince the world that the test is good enough to be used, to save babies.

## O4: Raising the profile of the placenta in perinatal healthcare delivery and research

### Raising the Profile of the Placenta in Perinatal Healthcare Delivery and Research

#### Mana M. Parast^1,2^

##### ^1^Department of Pathology, University of California San Diego, La Jolla, CA; ^2^Sanford Consortium for Regenerative Medicine, La Jolla, CA

*BMC Proceedings 2023,***17(14):**O4

The placenta is a transient organ, necessary for proper in utero growth and development. It has been called “the diary of intrauterine life,” yet remains the least-appreciated and, arguably, the least understood, of all human organs. On the clinical side, a complete gross and histopathologic examination of the placenta remains one of the simplest ways to gain insights into both maternal and neonatal complications of pregnancy, including preterm labor, fetal growth restriction, and stillbirth. Standardized guidelines for placental examination and sampling, as well as diagnostic criteria for placental lesions, were established by the Amsterdam Placental Workshop Group in 2016. This was recently followed by a proposed classification system, in which four major patterns of placental injury were defined, based on specific criteria, for ease of implementation of the Amsterdam Workshop guidelines. However, the lack of training programs in perinatal and placental pathology, superimposed on a similar lack of educational opportunities for both obstetricians and neonatologists in the applications of this information to, and implementation in, patient care, remain, among other factors, significant barriers to incorporation of placental evaluation in healthcare delivery. On the research side, scientists world-wide have made significant advances in establishing model systems for studying the human placenta. Specifically, since 2018, multiple groups have derived placental stem cells and “organoids” from early gestation tissues, while others have used embryonic/pluripotent stem cells to model this unique organ. The National Institute of Child Health and Human Development (NICHD) launched the Human Placenta Project in 2014; however, the limited funding made available (by comparison to other NIH programs, including the BRAIN Initiative), the increasing state and federal restrictions on use of early gestation placental tissues, as well as the lack of incorporation of placental histopathology into funded studies, continue to serve as barriers to further placental research, as well as to identifying evidence-based applications of placental evaluation to obstetric and neonatal healthcare. A coordinated effort by patients, healthcare providers, and researchers is required in order to raise the profile of the human placenta, and advocate for proper funding of basic, translational, and clinical placental research, lifting of restrictions on ethically-obtained placental tissue samples, and integration of placental evaluation into clinical trials carried out by the NIH-funded Maternal Fetal Medicine Unit (MFMU) and Neonatal Research Networks (NRN). This would be a significant investment into the health of, not just pregnant people today, but of many future generations to come.

## O5: Unsolved mysteries: Reinvestigating unexplained stillbirths for evidence of phasic uteroplacental hypoperfusion and its clinical associations

### Gar-Way Ma, Sarah Zachariah

#### University of Toronto, Toronto, Canada

*BMC Proceedings 2023,***17(14):**O5

**Background**

Stillbirth is a tragedy, and answers as to why the baby died are often elusive. While the placental pathology and fetal autopsy often yield diagnostic information regarding the cause, approximately 10-50% remain underexplained or unexplained, which does not allow for targeted interventions to reduce the risk of future occurrence. We hypothesize that some unexplained stillbirths occur in the setting of repetitive uteroplacental hypoperfusion events that occur overnight during intermittent and prolonged periods of maternal supine sleep when maternal arterial pressures are lower. To support our hypothesis, we expect that clinical risk factors for increased maternal venous pressures when supine (e.g., obesity, polyhydramnios, sleep apnea) and resultant uteroplacental hypoperfusion events will be associated with phasic-type neuropathological findings (e.g., hypoxic-ischemic encephalopathy as demonstrated by pontosubicular neuronal necrosis).

**Aims**

To identify correlations between clinically identifiable risk factors for increased supine maternal venous pressures with neuropathological findings associated with repetitive uteroplacental hypoperfusion events, allowing for stillbirth risk identification and prevention.

**Methods**

Fetal autopsies and placental pathologies from unexplained intrauterine fetal demises (IUFD) occurring between January 2005 through December 2021, inclusive, at Mount Sinai Hospital (Toronto, Canada) were reviewed. Cases were excluded if they occurred prior to 34 weeks' gestation; were terminations, intrapartum deaths, neonatal deaths, or multiple gestation; or had known severe maternal or fetal disease, major fetal visceral malformations, or aneuploidy. For included cases, we reviewed the fetal autopsy, placental pathology, and maternal clinical history. Descriptive and inferential statistical analyses were then completed.

**Results**

Of 3,196 stillbirths screened, 139 cases met the eligibility criteria. The lesions of interest, pontosubicular neuronal necrosis and chorangiosis, were found at a prevalence of 33.1% and 1.44%, respectively. Obesity and polyhydramnios were found in 7.91% and 4.32% of cases, respectively. There was only one case in which a diagnosis of maternal OSA was definitively documented in the clinical history. The results of multivariate logistic regression for associations between explanatory and response variables are pending.

**Conclusions**

The results of this study may lend insight into risk factors for late, unexpected, and unexplained IUFDs, which could have important implications for clinical practice and management in those identified to be at risk. Further, parents who have previously experienced a late, unexpected, and unexplained IUFD may find reprieve from the results of this study.

## O6: Congenital cytomegalovirus infection and adverse birth outcomes

### Michelle Zappas

#### Department of Nursing, University of Southern California, Los Angeles, California, USA

*BMC Proceedings 2023,***17(14):**O6

Cytomegalovirus (CMV), the most common cause of intrauterine infection and birth defects and has been associated with stillbirth. Classified as a DNA herpes virus family member, CMV is a beta herpesvirus, infecting most people during their lifetime. The virus is common, globally affecting 60-90% of the population but generally asymptomatic in those infected due to an individual's robust immune system. When symptomatic, a CMV infection causes cold-like symptoms such as sore throat, fever, fatigue, and lymphadenopathy; however, the symptoms are self-limiting and rarely cause concern in healthy children and adults.

If the mother acquires CMV during pregnancy, she may be unaware of it. Of the 30-50% of women of childbearing age in the United States who did not become infected in childhood or before pregnancy, about 1-4% become infected with their first CMV infection during pregnancy. Forty percent (40%) of these women pass the virus to the fetus, who is at higher risk for a symptomatic infection due to their immature immune system. Transmission to the fetus is 30-40% during the first and second trimesters but increases to 40-70% in the third semester. The most significant risk of complications occurs during primary infection in the first trimester for the fetus.

CMV infection initiates biomolecular changes in the placenta decreasing it's ability and resulting in IUGR. An overactive inflammatory response results in diminished trophoblast progenitor steam cells (TBPC) differentiation and extra villous trophoblast invasion. Fetal endothelial cells will increase collagen deposition resulting in fibrosis of the villous core. This remodeling of the placenta will result in restricted blood supply to the fetus, resulting in a hypoxic and stressful uterine environment. This may result in adverse birth outcomes following a CMV infection.

Congenital CMV infection recognition is often delayed by parents and providers. There currently is a vaccine in the works via Moderna, but until we are vaccinating women of childbearing age to help prevent transmission, education is key. Prevention strategies center on prenatal screening, handwashing and, hygiene.

## O7: Sleep disordered breathing is associated with hypoxia and alterations in placental apoptosis

### Ainslie Hancock^1^, Louise M. O’Brien^2^, Mana Parast^3,4^, Alexander E.P. Heazell^1,5^

#### ^1^Maternal and Fetal Health Research Centre, Division of Developmental Biology and Medicine, Faculty of Biology, Medicine and Health, University of Manchester, UK; ^2^Division of Sleep Medicine, Department of Neurology; Division of Maternal-Fetal Medicine, Department of Obstetrics & Gynecology, Michigan Medicine, Ann Arbor, Michigan, USA; ^3^Department of Pathology, University of California San Diego, La Jolla, CA; ^4^Sanford Consortium for Regenerative Medicine, La Jolla, CA; ^5^Saint Mary’s Hospital, Manchester University NHS Foundation Trust, Manchester, UK

##### **Correspondence:** E.P. Heazell

*BMC Proceedings 2023,***17(14):**O7

**Introduction**

Sleep-disordered breathing (SDB) describes a spectrum of respiratory abnormalities during sleep ranging from habitual snoring to obstructive sleep apnea (OSA). OSA may complicate 15-20% of pregnancies and increases the risk of gestational diabetes, preeclampsia, fetal growth restriction and preterm birth. The pathophysiology underpinning this association is poorly understood. This study aimed to determine whether maternal SDB is associated with morphological changes in the placenta.

**Methods**

Placental tissue was obtained from women who underwent polysomnography during pregnancy. These were grouped into: i) objectively-confirmed SDB (untreated); ii) objectively confirmed SDB (treated with positive airway pressure; PAP) and iii) women with no evidence of SDB. Participants were matched as closely as possible for gestational age, maternal age, ethnicity, body mass index and maternal cigarette smoking status. Semi-automated quantification was used following immunoperoxidase staining to count frequency and intensity of events including: cells in cycle (Ki67), apoptosis (M30 neoepitope), syncytial nuclear aggregates, vascularity (CD31), trophoblast area (CK7), oxidative stress (8-OHdG), hypoxia (HIF1α), macrophages (CD163) and leukocytes (CD45).

**Results**

There was an increase in HIF1α staining in women with SDB which was reduced to control levels in women who used PAP (Control Median 8.6 (IQR 4.0-10.6) vs SDB 10.8 (6.8-13.0) vs PAP 4.4 (1.3-5.8), p=0.03). HIF1α was expressed within trophoblast and Hofbauer cells. Levels of apoptosis were low in placentas but were increased in women using positive airway pressure (PAP) compared to controls (Median 0.02% (IQR 0.01-0.03%) vs 0.02% (0.02-0.04%) vs 0.03% (0.03-0.05%), p=0.04). There were no alterations in the number of cells in cycle, syncytial nuclear aggregates, vascularity, trophoblast area, macrophages or leukocytes between the different groups. Histopathological analysis found increased evidence of fetal and maternal vascular malperfusion in women with OSA.

**Conclusions**

Despite being associated with increased expression of HIF-1α, SDB was associated with little alteration in placental structure, contrary to what is seen in models of hypoxia in vitro. Further research is required to understand the pathophysiological processes underpinning the epidemiological association between SDB and pregnancy complications.

Funded by a grant from Star Legacy Foundation, MN, USA.

## O8: Does maternal sleep influence fetal growth?

### Louise M. O'Brien

#### Division of Sleep Medicine, Department of Neurology; Division of Maternal-Fetal Medicine, Department of Obstetrics & Gynecology, Michigan Medicine, Ann Arbor, Michigan, USA

*BMC Proceedings 2023,***17(14):**O8

Fetal growth restriction (FGR) is associated with increased perinatal morbidity and mortality including preterm birth, admission to the NICU, stillbirth, and long-term morbidities such as neurodevelopmental impairment and cardiovascular dysfunction. FGR is often the result of placental insufficiency and presents a major challenge in perinatal healthcare. There currently are no effective clinical interventions that improve intrauterine growth. Maternal sleep disordered breathing (SDB) has emerged as a predictor of poor maternal outcomes. Symptoms of SDB increase during pregnancy and affect up to 35% of women in the 3rd trimester and up to 85% of women with hypertensive disorders of pregnancy. Objectively-diagnosed SDB has been found in 10-25% of all pregnant women and approximately half of hypertensive pregnancy women.

Maternal SDB has been associated with fetal growth problems, including large for gestational age as well as FGR. Nonetheless, few studies have longitudinally investigated fetal growth across pregnancy. We have shown that while there were no differences in the proportion of infants with birth weight <10 th centile between women with and without objective measures of SDB, the presence of maternal SDB was predictive of a slowing in fetal growth in the 3rd trimester (aOR 3.6, 95% CI 1.4–9.4). Notably, women who had been treated with positive airway pressure during pregnancy did not demonstrate impaired fetal growth. In addition to growth measures, another indicator of fetal wellbeing is fetal heart rate pattern. We have also demonstrated that the majority (84%) of fetal heart rate decelerations are associated with maternal respiratory events. In women with prolonged fetal heart rate decelerations (mean duration of 4.5 mins), 90% of decelerations were temporally linked with maternal respiratory events, and all women had objective evidence of SDB. In summary, these data suggest that maternal SDB influences fetal wellbeing. Moreover, treatment of SDB may offer a clinical intervention that could improve fetal growth trajectory.

## O9: Positional therapy: A decade-long, multi-disciplinary, international collaboration for safer sleep in pregnancy

### Allan Kember^1^, Jane Warland^2^

#### ^1^University of Toronto, Toronto, Canada; ^2^School of Nursing, Curtin University, Perth, Australia

##### **Correspondence:** Allan Kember

*BMC Proceedings 2023,***17(14):**O9

**Background**

When Tomasina Stacey (New Zealand) published a landmark case-control study showing an association between supine sleep and late stillbirth in The BMJ in 2011, it did not take long for Louise O'Brien (USA), her student Jocelynn Owusu, and Jerry Coleman (Ghana) to complete a cross-sectional study in the West African context. It was their publication in 2013 that caught the attention of an engineer, Allan Kember (Canada), and prompted him to link up with Jerry, Louise, and Louise's colleague, Jane Warland (Australia). In 2013, the team invented the first positional therapy device for pregnancy (PrenaBelt) and, over the last ten years, have had an incredible journey together from clinical trials through commercialization.

**Aims**

Trace the research and development of the first positional therapy device for sleep during pregnancy from ideation through clinical studies and commercialization. Summarize the evidence for positional therapy during sleep after 28 weeks' gestation including the results of a recent Bayesian re-analysis.

**Methods**

In three tertiary-level centers, the team completed a randomized sham-controlled trial, a randomized sham-controlled cross-over trial, and a randomized controlled cross-over trial and published this work in peer-reviewed journals. A Bayesian re-analysis of the randomized sham-controlled trial was also completed in light of a recent individual patient data meta-analysis by Ngaire Anderson and colleagues (JAMA, 2019) and is currently under review. The team's engineers conducted dozens of user interviews, several rounds of beta testing, and in-house use tests to optimize the design based on user feedback. Hundreds of prototypes and research samples were iteratively produced. Along the way, challenges were encountered and overcome through multidisciplinary collaboration.

**Results**

In the sleep laboratory and in the home setting, over 240 participants in their third trimester have participated in the clinical research anywhere from two nights to twelve weeks, which amounts to nearly 17,000 nights of study in total. The device has been shown to significantly reduce the percentage of time sleeping on the back in the third trimester without affecting sleep quantity or quality. In the Bayesian framework, maternal use of the device during sleep throughout the third trimester has a high probability of benefitting the infant's birthweight centile by more than 5%. In a subset of participants with obstructive sleep apnea, the device significantly improved maternal respiratory parameters and fetal heart rate.

**Conclusions**

It is possible for passionate researchers and clinicians, through multi-disciplinary collaboration, to translate their discoveries into every-day solutions for patients.

## O10: Using fetal and infant mortality review (FIMR) to understand and catalyze action to reduce stillbirths

### Rosemary Fournier

#### Fetal and Infant Mortality Review, National Center for Fatality Review and Prevention, Michigan Public Health Institute, Okemos, Michigan, USA

*BMC Proceedings 2023,***17(14):**O10

COVID-19 is associated with increased risk for adverse pregnancy outcomes, including stillbirth. The CDC reports that 1,249,634 delivery hospitalizations during March 2020–September 2021, U.S. women with COVID-19 were at increased risk for stillbirth compared with those who did not have COVID-19.

The Fetal and Infant Mortality Review (FIMR) program is a public health surveillance tool that seeks to understand systems gaps contributing to fetal and infant deaths through comprehensive, multidisciplinary review of individual cases. This results in a community-driven approach to improved care, services, and resources for pregnant and birthing persons and families.

FIMR teams are conducting comprehensive reviews on cases of stillbirths and infant deaths that were impacted by COVID-19. FIMR teams are able to identify and address barriers families may have experienced during the pandemic by examining contextual factors and conducting parental or family interviews. Contextual factors explored during reviews includes the impact of changes in the rhythm of everyday life, changes in the ways agencies and providers delivered services, changes in the ways in which families accessed services, limitations to what services were available, and the increases in stress, and economic instability, and isolation.

Data from the National Fatality Review-Case Reporting System (NFR-CRS) was collected by over 100 FIMR teams during the pandemic. From 2000 to April 2023, 1,778 fetal deaths have been entered into the NFR-CRS. Of those, 191 (11%) identified that the death was impacted by COVID-19, and in 129 cases (68%) the childbearing parent contracted COVID-19 during pregnancy. Compared to mothers who did not contract COVID-19 and suffered a fetal loss during the same time period, mothers who contracted COVID-19 during pregnancy were more likely to have chronic hypertension, eclampsia, clotting disorders, oligohydramnios, and other placental abnormalities. In addition to the differences in medical conditions, detailed data is collected around system-level findings that illustrate gaps or needs for improvement in service delivery systems and social service programs. Examples of innovative prevention programs, driven by fatality review findings, will be shared.

FIMR teams offer a unique strategy for analyses of individual and community factors that significantly affect health disparities and are not discoverable through analyses of vital statistics and population-based data. FIMR, with its in-depth exploration and identification of factors that contribute to poor MCH outcomes, provides great insight into barriers families face in seeking and obtaining healthcare, and significant information used to drive strategies for prevention of stillbirths. This is particularly needed during the uptick in stillbirths related to COVID-19 infection.

**Acknowledgement**

The National Center is funded in part by Cooperative Agreement Number UG728482 from the U.S. Department of Health and Human Services (HHS), Health Resources and Services Administration (HRSA), Maternal and Child Health Bureau (MCHB) as part of an award totaling $5,149,996 annually with 0 percent financed with non-governmental sources. These contents are solely the responsibility of the authors and should not be construed as the official position or policy of, nor should any endorsements be inferred by HRSA, HHS or the U.S. Government.

## O11: Findings on the impact of the Covid-19 pandemic on maternity care access and fetal movement reporting from COSMOS (Covid-19: Outcomes for Sleep, Maternity care and glObal pregnancy Study)

### Louise O’Brien^1^, Isabelle Greatholder^2^, Alexander Heazell^2,3^, Stuart Watson^4^, Lindsey Wimmer^5^, Shauna Libsack^5^, Jane Warland^4,6^

#### ^1^Division of Sleep Medicine, Department of Neurology; Division of Maternal-Fetal Medicine, Department of Obstetrics & Gynecology, Michigan Medicine, Ann Arbor, Michigan, USA; ^2^Fetal and Infant Research Center, University of Manchester, Manchester, England; ^3^Saint Mary’s Hospital, Manchester University NHS Foundation Trust, Manchester, UK; ^4^King Edward Memorial Hospital, Perth, Australia; ^5^Star Legacy Foundation, Eden Prairie, Minnesota, USA; ^6^School of Nursing, Curtin University, Perth, Australia

##### **Correspondence:** Louise O’Brien

*BMC Proceedings 2023,***17(14):**O11

**Background**

The Covid-19 pandemic impacted global sleep health. In pregnancy, disrupted sleep is associated with adverse maternal-fetal outcomes, yet little is known about how the pandemic has affected sleep in this population.

**Methods**

An international survey of pregnant women was conducted online between April 2020 and October 2022. As part of the survey, women were asked about sleep habits before and during the Covid-19 pandemic and whether they delayed or altered presentation with reduced fetal movements (RFM).

**Results**

Overall, 2,171 ethnically diverse pregnant women completed the sleep questions. Median age was 31-years; 37% were Caucasian, 19% Asian, 12% Black, and 25% Hispanic/Latino. Median sleep duration did not change pre-Covid-19 to during Covid-19 (8-hours vs. 8-hours) although women reported waking approximately 45 mins later. Half (51%) said it was harder to get to sleep since the pandemic and worse sleep quality was reported by 54%. Those who found it harder to get to sleep, compared to those without change, were more likely to feel depressed (20% vs. 13%, p<0.001). Of the 4% of women who reported an abusive relationship; 69% found it harder to get to sleep during the pandemic (vs 49% of other women; p=0.001). The proportion of women experiencing racial discrimination in the healthcare setting doubled during Covid-19 (2% to 4.5%) and those experiencing discrimination were more likely to have insomnia symptoms (67% vs. 51%, p=0.04).

Women were more likely to delay or avoid care with RFM when they identified as Asian (adjusted Odds Ratio (aOR) 1.97, 95% CI 1.36-2.86), Black or African American (aOR 2.02, 95% CI 1.34-3.06) or more than one race (aOR 1.97, 95% CI 1.24-3.11). Women with high-risk pregnancies (aOR 1.82, 95% CI 1.38-2.41) and those who were multi-parous (aOR 3.12, 95% CI 2.25-4.33) were more likely to delay or avoid care after RFM (aOR 3.12, 95% CI 2.25-4.33). Reduced in-person visits (aOR 3.39, 95% CI 2.41-4.78), attending appointments alone (aOR 1.30, 95% CI 1.03-1.63) and Covid-19 concerns about healthcare services (aOR 1.67, 95% CI 1.29-2.18) also increased the likelihood of delay or avoidance following RFM.

**Conclusions**

The Covid-19 pandemic has negatively impacted the sleep of pregnant women. Given the known relationships between poor sleep and adverse pregnancy outcomes, the increase in sleep disruption in this population is of concern.

Alternations to the provision of healthcare services during the Covid-19 pandemic has significantly affected women’s engagement with services after RFM, increasing pre-existing global inequalities between women.

## O12: Fetal movements and stillbirth research: What we know and where to next?

### Billie Bradford

#### Department of Obstetrics & Gynaecology, Monash University, Melbourne, Australia

*BMC Proceedings 2023,***17(14):**O12

Title: Fetal Movements and Stillbirth Research: What do we know and where to next?

Stillbirth is devastating for parents and families. Predicting stillbirths on the basis of risk factors is not possible, and the cause of many stillbirths remains unexplained, even when thoroughly investigated. As long ago as the 1970s, it was observed that mother’s kick counts dropped significantly prior to fetal death in high-risk pregnancies. This observation led to trialing of various approaches to maternal monitoring of fetal movements to reduce stillbirths. Decades of research has yet to identify a method of fetal movement counting or promotion of fetal movement awareness that is effective in reducing stillbirths. Regardless a great deal has been learned along the way. Fetal movement counting promotes maternal-fetal attachment and fetal movement awareness interventions may lead to improved neonatal outcomes. Understanding of maternally perceived fetal movements in pregnancies with stillbirth has also improved.

Case-control studies have highlighted the features of normal fetal movements perceived by women in the third trimester. These include increasingly strong movements, increased frequency, fetal hiccups, evening movements, and multiple episodes of unusually vigorous movements. Conversely, fetal movement features associated with stillbirth include decreased frequency, decreased strength, a fetus that is quiet in the evening, and a single episode of unusually vigorous movement. Interestingly, risk of stillbirth may be highest in those with decreased frequency of fetal movements in early third trimester. Thus highlighting the need to advance techniques for surveillance of fetal and placental health.

Although high-quality evidence to guide management of fetal movements is lacking, maternal perception of decreased fetal movement remains an indicator of fetal compromise that demands thorough evaluation. Research to date has yet to provide clear guidance as to how we can harness women’s attention to and knowledge of their babies’ movements to reduce stillbirths. However, the importance of this aim has not diminished.

## O13: Verbal information from parents about stillbirth (the VIP study): Phase one

### Azriel Gan Lin Lo^1^, Lauren Breen^2^, Zoe Bradfield^1,3^, Scott White^3^, Sonya Criddle^3^, Georgia Griffin^3^, Bligh Berry^4^, Jane Warland^1,3^

#### ^1^School of Nursing, Curtin University, Perth, Western Australia, Australia; ^2^School of Population Health, Curtin University, Perth, Western Australia, Australia; ^3^King Edward Memorial Hospital, Perth, Western Australia, Australia; ^4^PathWest, Perth, Western Australia, Australia

*BMC Proceedings 2023,***17(14):**O13

**Background**

The cause of death (COD) of a baby following stillbirth is often determined based on case notes and pathology investigations in many countries. However, parents’ understanding of their baby’s COD can potentially impact or alter the diagnosis. The objective of this study was to develop a standardized interview schedule in collaboration with parents and other experts to allow them to contribute information that can enhance the overall understanding of stillbirth.

**Methods**

We conducted two rounds of a Delphi consensus process to develop the interview schedule. The Delphi panel consisted of bereaved parents, clinicians, and researchers recruited internationally. In the first round, each panelist was asked to provide up to five questions that they believed would help understand the COD in stillbirth cases. The questions were then categorized. In the second round, the panelists were asked to rate the importance of each category on a 4-point Likert scale, ranging from “not important to ask” to “vital to ask.” Consensus was considered achieved if more than 50% of the panelists rated a category as important or vital to ask.

**Results**

A total of 126 panelists participated in the first round, and 75 panelists participated in the second round. The majority of the panelists were bereaved parents (over 84% in each round). In the first round, 553 potential interview questions were generated. These questions were then grouped into sub-categories, including General Pregnancy Experience, Antenatal Care, Fetal Wellbeing, Maternal Wellbeing, Days prior to Stillbirth, and Perceived COD. In the second round, all of these categories reached consensus for inclusion in the final interview schedule, with positive consensus percentages ranging from 83% to 98%.

**Conclusions**

The inclusion of bereaved parents as the primary members of the panel provides a clear indication of the questions parents want to be asked. The finalized interview schedule will soon be tested with recently bereaved parents in an Australian Setting. The findings of this study will contribute to future research on how to incorporate parents’ perspectives in determining the cause of death in stillbirth cases.

## O14: Umbilical cord abnormalities in stillbirth: A pathologist’s perspective

### Linda M. Ernst

#### Department of Pathology, NorthShore University Health System, Evanston, Illinois, USA

*BMC Proceedings 2023,***17(14):**O14

The umbilical cord is an important physical connection between the placenta and the fetus and is composed of two fetal arteries and one large fetal vein surrounded by a cushioning substance known as Wharton’s jelly. Uninterrupted blood flow from the placenta to the fetus and vice versa depends on the umbilical cord, and so any disturbance in either the umbilical cord anatomy or its ability to have continuous blood flow can be detrimental to the fetus and potentially lead to stillbirth. Some of the anatomical abnormalities of the umbilical cord include excessive length, abnormal insertion to the placenta, excessive coiling, and the presence of a true knot. Other physical circumstances that can impede blood flow include body looping or other mechanical impingement of the umbilical cord.

Histologic criteria to establish umbilical blood flow obstruction as a cause of death in stillbirth can be used by pathologists, and the use of the initial causes of fetal death (INCODE) system to classify stillbirth related to umbilical cord abnormalities can be helpful in defining objective criteria. Full autopsy and placental examination are typically needed to classify an “umbilical cord accident” as the cause of death since other intrinsic fetal causes must be excluded.

Research has shown that obstruction of fetal blood flow related to umbilical cord abnormalities is a common cause of death in both the second and third trimester fetus and that hypercoiling of the umbilical cord is one of the most common anatomic abnormalities. Umbilical cord coiling patterns associated with the most significant compromise of the umbilical cord, the segmented and linked patterns, are also associated with stillbirth, as is the right coiling pattern. While umbilical cord abnormalities underlie a significant number of stillbirths, there are no reliable clinical risk factors to predict these outcomes. Further research is needed to find pregnancies at risk for stillbirth due to umbilical cord abnormalities.

## O15: Obstetric outcomes among women with reactive hypoglycemia at glucose tolerance test

### Sana Rehman, Salsabeel Kazi, Nicola Wallis, Lindsay Kindinger, Melissa Whitten, Dimitrios Siassakos

#### University College London Hospital, Beckenham, Kent, England

##### **Correspondence:** Sana Rehman

*BMC Proceedings 2023,***17(14):**O15

**Background**

Gestational diabetes (GDM) affects 1:20 pregnancies in the UK and is associated stillbirth. Women at risk are screened between 24-28 weeks. Some types of glucose dysregulation may remain undiagnosed and consequently at risk of adverse outcomes.

**Aims**

To assess obstetric outcomes in antenatal women with 'reactive hypoglycemia' (RH) during an oral glucose tolerance test (OGTT), defined as a 2-hour blood glucose level lower than the fasting value.

**Methods**

A cross-sectional study of 1063 pregnant women attending University College London Hospital for an OGTT between April 2019-July 2020. Outcomes were compared across three groups: RH, GDM and controls.

**Results**

In the sample, 301 women had RH, 450 had GDM, and 312 had normal OGTT (controls). Mean birth weight was greatest in the RH group (3355±539g) compared to both the GDM (3208±575g, p=0.0002) and control groups (3298±522g). Relative to controls, RH had higher rates of polyhydramnios (7.8% vs 3.9%, p=0.03), abdominal circumference >95th centile (11% vs 5.1%, p=0.005), small-for-gestational age infants (5.3% vs 2.4%, p=0.048) and neonatal oxygen desaturation (1.3% vs 0%, p=0.04). Compared to GDM, RH babies were more likely to have ambiguous genitalia (2.5% vs 0.4%, p =0.01). Rates of chorioamnionitis were higher in RH (4.6% vs GDM 2.7% vs controls 1.9%) as was gestational hypertension (12.3% vs 10.5% vs 10.6%, respectively). There were stillbirths only in the RH and GDM groups (RH n=1, GDM n=3, controls n=0).

**Conclusion**

Women with RH in this sample were at risk of adverse outcomes usually associated with insulin resistance and diabetes. Whilst the association with stillbirth requires further research, our clinical experience suggests that utilizing RH as an indicator may help to further understand and prevent unexplained stillbirths.

## O16: Specialist care in pregnancies after loss: An update from Rainbow Clinic, Manchester

### Emilie Bailey,^1^ Kajal Tamber,^2^ Rebecca Barron,^1^ Emma Tomlinson^1^, Alexander Heazell^1,2^

#### ^1^Saint Mary’s Hospital, Manchester University NHS Foundation Trust, Manchester, UK; ^2^Maternal and Fetal Health Research Centre, University of Manchester, Manchester, UK

##### **Correspondence:** Alexander Heazell

*BMC Proceedings 2023,***17(14):**O16

**Background**

In 2013, the Rainbow Clinic was established in Saint Mary’s Hospital, Manchester, UK to provide care for women in pregnancy after stillbirth. Since then it has provided care for over 1,000 families with the model of care being refined. Following successful evaluation of this clinic a decision was made to disseminate the model of care to other UK maternity units. Patient experience has continued to be evaluated in the Rainbow Clinic to ensure that care provided meets parents’ needs.

**Methods**

To disseminate the Rainbow Clinic model, we initially developed a formal pathway towards opening Rainbow Clinics. We commenced The National Rainbow Clinic study to record the outcomes of pregnancy for women and their babies and will assess the psychological outcomes for women and record their experiences of attending Rainbow Clinic. This evaluation commenced in the summer of 2021 and is planned to run in at least 20 sites until 2026.

We developed a 13-item questionnaire developed with input from service users. It was distributed to pregnant women who attended the Rainbow Clinics in Manchester between July 2016 and June 2021. The questionnaire was completed at the last appointment before planned birth. Descriptive statistics and unpaired t-test were used for quantitative data and summative content analysis for qualitative data.

**Results**

There are now 26 active Rainbow Clinics distributed over a wide geographical area. We now have over 1,000 women who have started the Tommy’s Rainbow Clinic Study and outcome data on 595 participants. Using the data supplied from participating units, we have developed and tested a dashboard which allows comparison between sites and assessment of whether the Rainbow Clinic model is achieving improvements in outcomes.

456 women completed the questionnaire. The mean patient experience score per quarter was stable with an average of 21.1 (±3.0) out of 25. The COVID-19 pandemic had no effect on patient experience (pre-pandemic v during-pandemic: mean 21.2 v 21.3; p=0.75). Individually, all questions scored highly (mean scores 1.81-1.95 (±0.23-0.41) out of a maximum of 2) apart from the question regarding the ‘Rainbow sticker’ attached to antenatal notes to identify women who have previously experienced perinatal loss (1.51 ±0.79).

**Conclusion**

Specialist antenatal care provided by the Rainbow Clinic was rated as of a high standard which was viewed favorably by women attending. Potential future improvements include sticker alterations (or other mechanisms to identify women) and increased awareness of the clinic in other institutions.

## O17: Fetal Assessment: Past, Present, Future – A Journey to Reduce Stillbirth

### Lawrence D. Platt

#### Department of Obstetrics and Gynecology, David Geffen School of Medicine at UCLA; Center for Fetal Medicine & Women’s Ultrasound, Los Angeles, California, USA

*BMC Proceedings 2023,***17(14):**O17

Stillbirth is a global issue with 13.9/1000 total births reported in 2021, with the highest rates reported in India. More than 40 years ago, five fetal biophysical variables were identified (fetal breathing movements, fetal tone, qualitative amniotic fluid volume, and the nonstress test) and were used individually and in combination to determine their relationship to pregnancy outcome. The relationship between the variables, individually and in combination, to pregnancy outcome were judged by a 5-minute Apgar score, fetal distress in labor, and perinatal mortality rate. Individual variables revealed a low false negative rate and was similar between tests while a higher false positive rate (>50%) varied significantly between tests. The initial findings lead to our development of a fetal biophysical profile. Using all five of the variables in fetal assessment provided the most accurate differentiation between a normal fetus and a compromised fetus.

Repeated studies have shown that the use of the fetal biophysical profile as will be demonstrated in this presentation has the ability to reduce fetal death when used appropriately. What does the future hold? Over the years we have seen ultrasound systems shrink in footprint from the large units (circa 1956) to the compact ones (circa 1975) to the handheld devices that are becoming commonplace today. This development has led many patients to use these handheld systems to replace their use of doptones. Artificial intelligence (AI) will continue to advance and improve handheld ultrasound systems. Ultrasound solutions will utilize open platforms enabling earlier integration with AI-powered innovations.

## O18: Using digital animation as a way of communicating public health messages to reduce stillbirth in linguistically diverse communities

### Tomasina Stacey

#### Kings College London, London, England

*BMC Proceedings 2023,***17(14):**O18

Pregnant women are presented with a wide range of information to help keep them and their babies safe in pregnancy and birth. There are important public health messages for women and birthing people to be aware of to reduce their risk of stillbirth. This information is often presented in such a way that it is not accessible to all. Research shows that giving important health information through digital animation helps people to remember and act on the messages. Digital animation is particularly good for those with low health literacy or who experience language barriers.

This presentation will discuss the complexity of communicating public health messages to help reduce the risks of stillbirth, making them accessible and not engendering anxiety. It will recap on some of the key areas of advice that have been identified as being of benefit and it will then describe the use of digital animation that is co-developed as a way to ensure that the messages are accessible to a culturally and linguistically diverse community.

To co-develop and evaluate a digital animation to increase knowledge and awareness of how to reduce stillbirth risk within an area with a multi-ethnic population.

The 2.3 minute animation about key stillbirth-prevention messages was co-designed with ethnic minority service users and midwives, and translated into multiple languages (English, Urdu, Arabic reflecting local demographics). The character in the animation, clothes, colours, other pictures, and words used were developed by the group to ensure they are sensitive and relevant.

## O19: The blame game: Experience and impact of stillbirth on midwives

### Tosin Popoola

#### School of Nursing and Midwifery, University of Newcastle, Australia

*BMC Proceedings 2023,***17(14):**O19

**Introduction**

After the diagnosis of a stillbirth, parents need supportive care from health professionals to deal with the loss. However, while health professionals are expected to provide appropriate bereavement care during and after stillbirth diagnosis, they also have their own grief and sadness to deal with. Lack of understanding of health professionals' experience of stillbirth can affect the quality of bereavement care they provide, which can lead to negative grief trajectory for parents. However, there is limited research on health professionals' experience of stillbirth and how it impacts them.

**Aim**

The aim of this study was to understand the experience of health professionals who have delivered a stillbirth in clinical practice.

**Methods**

Individual interviews were conducted with 11 registered midwives in Nigeria about their stillbirth experience. The interviews were analyzed thematically, and the analysis yielded four main findings.

**Results**

In the first theme, midwives discussed their emotional/psychological reaction to stillbirth, which includes self-blame, blaming others (even parents), guilt and emotional trauma. In the second theme, the midwives discussed the impact of stillbirth on their professional identity, which includes employers' scrutinization of their midwifery competence, violence from dissatisfied family members, loss of the 'joy of midwifery', loss of job satisfaction, and self-doubts about professional competence. In the third theme, the midwives discussed the impact of stillbirth on their practice, such as increased assisted births and caesarean sections due to fear. However, they also said they had more motivation to learn about stillbirth prevention after they experienced it. In the fourth theme, the midwives discussed their coping mechanisms, which they described as a combination of personal resilience, requesting day offs and seeking the support of colleagues to regain professional confidence.

**Conclusion**

As this study shows, stillbirth occurs within the context of culpability. As the health professional, family and the health system try to process the loss, the practice and professional identity of a health professional might be questioned and scrutinized. As a result, stillbirth increases the risk of second victim phenomenon for health professionals. This suggests that health professionals need more support to deal with the personal and professional impact of stillbirth before they can manage the grief of stillbirth-affected families appropriately.

## O20: Stigmatization through marketing and societal factors after baby loss: Understanding contributors to maternal mental health and well-being

### Elizabeth A. Minton

#### Department of Marketing, University of Wyoming, Laramie, Wyoming, USA

*BMC Proceedings 2023,***17(14):**O20

**Background**

Almost one in three women has suffered the loss of a child due to miscarriage, stillbirth, or infant death (i.e., what we refer to collectively as "baby loss"). Bereaved mothers who experience such loss face great stigma in their personal lives and in the marketplace as baby loss is a taboo topic that violates the natural order of life, and societal norms imply that this topic should not be discussed. This leads bereaved mothers to feel that their mother identity is stigmatized and devalued, thereby contributing to maternal health issues outside of purely the physical health outcomes of loss. Marketers routinely incorporate child-related messages in advertising tactics, such as asking questions about the number of children someone has on forms, asking parents to share a photo of their child, or posting advertisements depicting happy people in pregnancy. These actions can trigger feelings of grief and devastation for bereaved mothers.

**Aims**

In examining a consumer process model of stigmatized loss, our research aimed to identify what contributes to baby loss stigma, the factors that perpetuate this stigma, and what can be done by marketers, nonprofit organizations, and other entities to break down this stigma and allow bereaved mothers to freely experience their mother identity, bringing value to themselves as well as their deceased babies.

**Methods**

Through 30 qualitative interviews with bereaved mothers, we develop a model depicting the processes consumers go through in experiencing stigmatized loss.

**Results**

This research builds off the identity-threat model of stigma to showcase the process of stigmatized loss as well as brings these consumers what nearly all stated they need more of – acknowledgement. Key themes emerged as we explored stigmatized loss discourses including marketing serving as a situational cue that triggers stigma, identity-based responses trying to preserve both a baby and mother's identity, nonvolitional and volitional responses to seek control and reconstruct identity, positive and negative outcomes stemming from changing stigma and avoiding stigmatized identity activation, and identification of both positive and negative triggers that initiate a recursive process through stigmatized baby loss. Importantly, stigma can be perceived as both an identity threat (negative) and identity confirmation (positive).

**Conclusions**

This research area is ripe with opportunity for marketers, other support organizations, and individuals alike to help facilitate healing and well-being for bereaved mothers and others experiencing stigmatized loss. This healing is essential for maternal mental health outcomes.

## O21: Supporting bereaved parents experiencing pregnancy and infant loss: Skills development, research updates and caring for the bereaved

### Kathleen Massmann

#### Healing Moments Counseling, Monticello, Minnesota, USA

*BMC Proceedings 2023,***17(14):**O21

The prevalence of those who have experienced infant, pregnancy, and child loss is significantly greater than many people are currently aware of; in the United States, one in four women will experience miscarriage or stillbirth in their lifetime. After a loss, women's long-term psychological effects are often misunderstood, and often support is lacking; almost 20% of women who experience miscarriage show trauma, depressive, and/or anxiety-based symptoms. The research literature on bereaved parents indicated that infant, pregnancy, and child loss are traumatic grief. Yet, there are a limited number of providers, researchers, and professionals who specialize in this area.

## P1: Improving health professionals' knowledge and perception of maternal sleep position as a modifiable risk factor for stillbirth

### Lindsey J. Wimmer^1^, Barbara Toppin^1,2^, Nicole B. Beckmann^3^

#### ^1^Star Legacy Foundation, Eden Prairie, Minnesota, USA; ^2^Adefris & Toppin, Woodbury, Minnesota, USA; ^3^Department of Nursing, St Catherine University, St. Paul, Minnesota, USA

##### **Correspondence:** Lindsey J. Wimmer

*BMC Proceedings 2023,***17(14):**P1

**Background**

The silence surrounding stillbirth limits research and exacerbates the impact on families and health professionals. In the United States, the stillbirth rate has been unchanged for decades and demonstrates significant racial disparities. Maternal sleep position in the third trimester has recently emerged as a modifiable risk factor, yet it is not widely discussed amongst health professionals or pregnant women.

**Local Problem**

Focus groups indicated that health professionals do not utilize maternal sleep position as an intervention to reduce stillbirth. The most significant barriers noted were a lack of knowledge, fear of increasing patient anxiety, and limited provider time with pregnant women.

**Methods**

This project developed and implemented an online education module for physicians, nurse midwives, nurse practitioners, nurses, social workers, and doulas who work with pregnant women. Health professionals were given information about the project through a nonprofit stillbirth organization. The health professionals completed surveys before and after viewing the module and a final follow-up survey three months later.

**Intervention**

The educational module described the current evidence regarding the relationship between stillbirth and maternal sleep position in the third trimester, how to discuss these topics with pregnant women, and resources available for families.

**Results**

The health professionals demonstrated statistically significant improvements in their knowledge of stillbirth trends, racial disparities in stillbirth, and maternal sleep position for preventing stillbirth in the third trimester. Health professionals were more confident in implementing this concept into their practice and reported they intend to continue using the information in their practice.

**Conclusions**

Health professionals improved their knowledge about maternal sleep position as a modifiable risk factor for stillbirth and were more confident implementing it into practice after completing an online education module.

## P2: Facilitating support groups for bereaved families

### Joann O'Leary

#### Star Legacy Foundation, Eden Prairie, Minnesota, USA

*BMC Proceedings 2023,***17(14):**P2

This poster presentation will describe the use of support groups for families who have experienced the loss of a baby and in the pregnancy that follows. Content includes support at the time of loss, the pregnancy that follows, parenting/raising children who have experienced loss and grandparent/extended family members. It will discuss the literature on support groups for bereaved families, the pros and cons of involvement, and objectives of each group.

A common concern for the Pregnancy After Loss group include how to continue the bond with the deceased baby. Discussion centers around 1) the fetal cells of our children remaining in a woman’s body for many years after a loss, 2) the unborn baby in the womb is sharing the same space as deceased baby so already know the sibling, 3) parents don’t need to wonder when to share the story of the deceased sibling, 4) the deceased baby will always be a big sibling to future living children, and 5) how to protect themselves from others who do not understand.

In the Grandparents group, participants express appreciation for learning that they are not alone. Other frequent topics include 1) understanding that they are changed people, too, 2) holidays are difficult, 3) family dynamics can change drastically, and 4) how to be happy for a sibling having a new baby without hurting the bereaved parents.

## P3: The DOSAGE study protocol: Dose Of Supine sleep Affects fetal Growth? an exposure-response study

### Allan Kember^1^ Jane Warland^2^

#### ^1^University of Toronto, Toronto, Canada; ^2^School of Nursing, Curtin University, Perth, Australia

##### **Correspondence:** Allan Kember

*BMC Proceedings 2023,***17(14):**P3

**Background**

The Russo-Williamson thesis states that a causal hypothesis in medicine can be established only by using both statistical evidence and evidence of mechanism. In the last five years, evidence of mechanism between supine sleeping position and adverse pregnancy outcomes has been growing rapidly (e.g., work of Peter Stone et al.); however, the quality of statistical evidence has opened the door to criticism and skepticism. In 2021, the Royal College of Obstetricians and Gynaecologists reviewed the body of statistical evidence and noted a major weakness: all studies to date were retrospective and sleeping position was not objectively measured. As such, they concluded that it would not be possible to prospectively study the association between sleeping position and adverse pregnancy outcomes. Recently, we built a deep learning vision-based model (SLeeP AIDePt) for automated detection and quantification of maternal sleeping position across the third trimester of pregnancy in the home setting.

**Aims**

The aim of the DOSAGE Study is to gather objective evidence to either lend support to (or detract support from) a causal link between supine sleeping position after 28 weeks' gestation, fetal growth, and late stillbirth, and to quantify the safe "dose" of nightly supine sleeping time, if any.

**Methods**

We present a protocol for an international, prospective, cohort study to characterize the exposure-response relationship, if any, between sleeping position, fetal growth, and late stillbirth. Eligibility criteria includes ≥28 weeks' gestation, sleeps in a bed a night, possesses or can easily procure a home-security camera, and bed partner, if any, consents to participate. Participants will record their sleep one night per week from 28 weeks to birth using any commercially-available home-security camera mounted above the head of their bed. Participants will upload their videos to a secure, online portal where the SLeeP AIDePt model will automatically detect and quantify the amount of time spent in each sleeping position, which, averaged over approximately twelve weeks, will act as a surrogate of the average dosages of time spent in each sleeping positions per night across the third trimester. After birth, participants will self-report pregnancy, labor, and birth outcomes. The primary outcome, customized birthweight centile per GROW (Jason Gardosi, et al), will be computed and regressed on sleep position dosages. Stopping criteria will be employed per Bayesian methodology.

**Conclusions**

The DOSAGE Study is currently looking for collaborators and partners and is set to launch in September 2023.

## P4: Childbirth education for pregnancy after loss: Not just another birth

### Lindsey J. Wimmer, Jennifer Kouri

#### Star Legacy Foundation, Eden Prairie, Minnesota, USA

##### **Correspondence:** Lindsey J. Wimmer

*BMC Proceedings 2023,***17(14):**P4

**Background**

The majority of families who experience the death of a baby during pregnancy or infancy will have at least one additional pregnancy. These subsequent pregnancies are known to be highly stressful and traumatic processes for the entire family. As birth approaches, many parents feel their anxiety increasing as they prepare for the many emotions combined with their grief and trauma from the previous pregnancy. The physical preparation is also concerning because many families will expect or hope for a different type of delivery, have additional physical health concerns in this pregnancy or did not attend childbirth education classes before their previous delivery. Unfortunately, many families report feeling uncomfortable attending the childbirth education classes in their community because they have questions specific to their past experiences, and they realize that their history may be difficult for the other attendees to hear.

**Methods**

A national nonprofit organization presented a virtual childbirth education class specific to families experiencing a pregnancy after a previous loss. The classes are four hours in length, held over two sessions. The free, online format allows individuals from across the country to participate. Registration is limited at each offering to ensure families have adequate time to ask questions and share information about their previous pregnancies. A labor and delivery nurse with specialized training in perinatal loss teaches the sessions. Content includes birth planning, types of delivery, stages of labor, pain management, common emotions, self-care, postpartum care, tips for spouse/partner, coping with stress/anxiety, advocating for yourself, and available resources.

**Results**

To date, 347 families from 39 states, three Canadian provinces, and six countries have participated in the classes. Evaluation surveys show that families overwhelmingly find the courses helpful, and 100% of respondents said they would recommend the class to a friend. Comments are very positive and confirm that families appreciate being in a class with other families who have had similar experiences in their past pregnancies.

**Conclusion**

Childbirth education classes designed for families in pregnancies after a poor pregnancy outcome are desired by many families and found to be beneficial as they navigate the anxiety of this pregnancy and prepare for an emotional delivery. This program's strengths include the instructor's expertise, no cost to participants, and virtual format. Future initiatives will expand the number of offerings, provide sessions tailored to specific conditions, and sessions for non-English speaking families.

## P5: Centralizing resources for bereaved families

### Lindsey J. Wimmer, Kelly Pulford, Mikayla Allen, Denise Nelson

#### Star Legacy Foundation, Eden Prairie, Minnesota, USA

##### **Correspondence:** Lindsey J. Wimmer

*BMC Proceedings 2023,***17(14):**P5

**Introduction**

Perinatal loss is a devastating event that leaves many families to grieve in silence. The parents are at a higher risk for mental health conditions, financial difficulties, employment concerns, and relationship challenges. Unfortunately, many community services are under-utilized due to a lack of awareness, cultural mistrust, or the time and energy required to research options. Families also share that even when resources are known, it is emotionally challenging to reach out and ask a stranger for help.

**Aim**

The Minnesota Center for Stillbirth and Infant Death was created to be a centralized resource for families who experience the death of a baby during pregnancy or infancy. There is an emphasis on the health of the entire family, minimizing effort for the families, and being a long-term source of support and information.

**Methods**

Families are referred to the program by health professionals, community leaders, friends, or themselves. Fetal and infant death reports from the Minnesota Department of Health also serve as a referral source. Families are initially contacted with a sympathy card in the mail. A staff member then reaches out to the family by text, phone, or email to offer support. Common activities include listening to their baby's story, validating their emotions, and referring them to community resources such as counseling, support groups, peer support, and financial assistance. Follow-up letters are sent three, six, and 12 months after the baby's death and as needed by the family. Families are reassured that they may also contact the program as needed.

**Results**

The Minnesota Center for Stillbirth and Infant Death launched in the fall of 2019. Through April 2023, 2,077 families have been referred to the program. Of those, 69% have utilized the services. Each year, the percentage of referrals from the community increases, allowing the death reports to serve as a backup rather than the primary referral source. Families report satisfaction and gratitude for the support and information. In January 2023, the program was expanded to offer the same services to grieving families in Wisconsin.

**Conclusion**

The Minnesota Center for Stillbirth and Infant Death is a successful community support program serving families who have experienced the death of a baby during pregnancy or infancy. By offering a centralized service, families are more likely to engage with appropriate resources and receive the needed support.

## P6: Screening past reproductive losses

### Angelica Quezada, Cara Buskmiller, Kathryn R Grauerholz, Jennifer Bute, Maria Brann, Michaelene Fredenburg, Jerrie Refuerzo

#### Institute of Reproductive Grief Care, San Diego, California, USA

##### **Correspondence:** Angelica Quezada

*BMC Proceedings 2023,***17(14):**P6

Reproductive loss such as stillbirth can result in prolonged grief. In many cases this grief remains unacknowledged by healthcare providers resulting in disenfranchised grief and in 15-25% of pregnancy loss complicated grief reactions. Our physicians, midwives, nurses, social workers and families have been ill equipped with brief screening tools focused on examining other mental health needs such a perinatal mood and anxiety disorders, failing to assess for complicated grief reactions.

We have collaborated with the University of Texas Health Science Center at Houston, and have a preliminary validation of the Reproductive Grief Screen (RGS). This is a validated five question screening tool which can be implemented in an array of multidisciplinary practices for identifying prolonged and maladaptive grief reactions allowing healthcare providers to appropriately and promptly refer or provide direct grief care.

A Reproductive Grief Screen (RGS) based on the BGQ was crafted by a team of two psychologist, one nurse, and one was conducted with 140 women who reported experiencing a reproductive loss. Reproductive loss was defined as a miscarriage, stillbirth, neonatal death, infant death, selective reduction, or termination of pregnancy. These individuals were provided with three additional validated perinatal grief assessment surveys (PGIS, PDSS, and IESR) to investigate the ability of the RGS. Descriptive statistics were calculated for each of the surveys provided, a Cronbach's alpha coefficient was calculated, along with a confirmatory factor analysis to assess factorial validity of the RGS.

The survey met Fornell and Larker criteria and found a high correlation between RGS and PGIS suggesting measurements of the same perinatal grief that previously validated, longer and not often used instruments. The majority of participants (51%) had a score of 4 or more, indicating a positive screening test. The weakness of the study conducted with UTHealth Houston, is the variety of demographic representations, making it one of the reasons we need to have healthcare providers in multidisciplinary settings and with a broader population implement this screening tool.

Additional research has been conducted with women who have experienced reproductive loss at IUPUI, to learn from individuals what would meet their needs regarding grief care. Concluding that not only do we need to assess for reproductive loss, understand how to implement screening tools such as RGS, but there is also the need to transform reproductive grief care language to promote preferred communication practices with patients and their families as they receive compassionate client centered grief care.

## P7: Pregnancy research project: Creating a comprehensive view of pregnancy

### Lindsey J. Wimmer

#### Star Legacy Foundation, Eden Prairie, Minnesota, USA

*BMC Proceedings 2023,***17(14):**P7

**Introduction**

Progress in stillbirth prevention and care has been limited for decades in part due to the lack of research. Researchers indicate that frequent barriers include the cost, relatively low-frequency in single institutions, and stigma experienced by researchers, health professionals, and families. Additionally, most studies consider information from the medical records or the pregnant women, but not both.

**Aim**

An international team of stillbirth researchers collaborated to design a research program that will allow enrollment from around the world and evaluate multiple research questions. It includes objective information from medical records and connects the data to responses from a maternal survey to provide a robust, holistic view of pregnancy health. Participants enroll once, but their information may be utilized by dozens of research studies. Outside researchers with IRB-approved studies will also be able to request de-identified data points.

**Methods**

Participants will include individuals who are at least 18 years old and are English-speaking. They may participate once for each eligible pregnancy. Pregnancies include current pregnancies, any pregnancy in the last five years, or any pregnancy that ended in stillbirth. Recruitment is occurring through a nonprofit organization serving bereaved families and partnering organizations who also engage with pregnant or recently pregnant women. Participants complete an online survey about their pregnancy experience, including details about medical and family history, fetal movement, maternal sleep and activity, social factors, and other factors felt to influence the pregnancy outcome. By signing a request for medical records, they consent for their health providers to send their records to the study. Trained abstractors collect data from the records and connect them with survey responses in the database. Participants can also indicate their interest in other studies.

**Current Status**

Enrollment is active and recruitment activities are being implemented. The consent form and survey will soon be available in Spanish. Initial analysis will begin when 5,000 participants have completed the project.

**Conclusion**

The Pregnancy Research Project is a study designed by an international team of researchers to address some of the barriers to research aimed at stillbirth prevention and care. It has a unique approach that allows multiple study questions to be considered, includes objective and subjective data, and allows participation from around the world. This project has the potential to significantly increase what is known about promoting healthy pregnancies and better serving bereaved families.

## P8: White tears: Lactation and pregnancy and infant loss

### Brandy Gentry

#### BirthRoot Community Doula Alliance, Erie, Pennsylvania, USA

*BMC Proceedings 2023,***17(14):**P8

**Introduction**

Lactation and bereavement, due to pregnancy or infant loss, in the medical field may appear to be feel very separate occurrences to the provider in practice, however, they are fully intertwined experiences for the woman who is bereaved and is who experiences pregnancy or infant loss. Many women are unaware when they begin to lactate, and few are aware or supported by their providers with the range of individualized options regarding suppression, expression, or donation.

**Aims**

The aim of this presentation is to provide an overview of practical evidence that bereavement and lactation are inseparable experiences. This will be achieved by exploring the physiologic experience of childbirth and lactogenesis, the psychologic experience of childbirth and lactogenesis, and the emotional and spiritual experience of childbirth and lactogenesis.

**Methods**

Provide evidence through an interactive discussion to support the implementation of best care practices in care in order to better inform and prepare providers to increase support for the women experiencing bereavement and lactogenesis.

**Conclusion**

When lactogenesis and pregnancy and infant loss are acknowledged as an inseparable experience, bereaved mothers have better physical, psychologic, and emotional and spiritual outcomes. Lactogenesis and pregnancy and infant loss are inseparable experiences and intentional, comprehensive, compassionate care must be provided for better outcomes to occur.

## P9: Perinatal mental health, bonding, and attachment: How to grow a rainbow

### Katherine Hyde-Hensley

#### Star Legacy Foundation, Eden Prairie, Minnesota, USA

*BMC Proceedings 2023,***17(14):**P9

Pregnancy after a stillbirth is a delicate time for a bereaved parent. Nesting Circles offer a comprehensive psychoeducational approach to supporting maternal mental health needs during the subsequent pregnancy. This innovative model supports evidenced-based attachment resources with the peer-to-peer community of shared experiences. The goal is to support the family with a positive prenatal experience and postpartum resources for delivering a rainbow baby.

## P10: Pregnancy after loss app: Week-by-week support for PAL parents in their pockets

### Lindsey M. Henke

#### Pregnancy After Loss Support, Bloomington, Minnesota, USA

*BMC Proceedings 2023,***17(14):**P10

**Background**

Pregnancy After Loss Support is dedicated to ensuring that every parent who is experiencing pregnancy after loss is able to find support and connection among both peers and health care professionals who understand and validate the unique and complex experience of pregnancy after a previous perinatal or child death. Pregnancy After Loss Support (PALS) strives to support parents pregnant after a loss and encourage them to choose hope over fear while nurturing grief during their subsequent pregnancy. PALS accomplishes this goal through services for parents pregnant again after a loss and the providers who support them. PALS services include an online magazine, online peer-moderated support groups, local meet-ups, outreach and education, and most recently the first of its kind and only week-by-week Pregnancy After Loss App specifically designed for the pregnant after loss parent.

**Methods**

The first app for parents who are conceiving or pregnant after loss - brought to you by Pregnancy After Loss Support (PALS). It's designed to support the entire emotional journey of parents as they go through pregnancy with potential ups and downs of their experience in mind. There are over one million women who experience pregnancy loss each year in the United States and 50-80% of them will conceive after a loss within 12-18 months of their previous pregnancy loss. This is the first app for their unique journeys. The PAL App Includes: Week-by-Week evidence-based updates about the pregnancy progress that are tailored to the unique emotional experience of experiencing both the joy and grief during the pregnancy that follows a loss. Psychoeducation tailored to the individual user's experience including targeted support around anniversaries of losses in the calendar year and week of gestation loss information from past pregnancy as it relates to current pregnancy. Twenty plus coping skills to prepare for and persevere through hard moments that accompany a pregnancy after a loss. Ten meditations offered to help the pregnant after loss parent calm their nervous system during the stressful moments of pregnancy after loss. Access to online communities in the app and through Pregnancy After Loss supports fourteen plus online peer-to-peer support groups that address topics that are unique to the experience of pregnancy after miscarriage, stillbirth, and infant death. Weekly content and articles written by or approved by health care professionals that relate to the traditional milestones in a pregnancy as well as those that relate to the unique milestones of a pregnancy that follows a loss. Grief and loss, trying to conceive, and postpartum (parenting after loss) psycho-education resources as they relate to the parent pregnant after a loss.

**Results**

15,570 downloads since September 2021. Access: Free and downloadable on both Apple App Store and Google Play. Overall Satisfaction of App (out of 100 survey participants as of 12/2022): 92% report being very satisfied/somewhat satisfied with Pregnancy After Loss App. 0% report being dissatisfied with the Pregnancy After Loss App.

## P11: Connecting with fetus: The use of app-based fetal movement counting and experiences during pregnancy and birth

### Olivia Ceavers

#### Florida International University, Miami, Florida, USA

*BMC Proceedings 2023,***17(14):**P11

**Introduction**

Studies suggest that stillbirth may be reduced when uniform fetal movement monitoring is introduced, and pregnant people are often introduced to fetal movement counting to increase awareness of changes in movement beginning in the third trimester. As use of pregnancy-related mobile applications (apps) increased in the past decade, several have incorporated a "kick counter" that allows for convenient fetal movement counting. This study aims to examine the impacts of app-based fetal movement counting on experiences during pregnancy and birth.

**Method**

This study used two types of secondary data including the consumer's app use and their end of pregnancy survey data collected by Healthy Birth Day Inc., the provider of the Count the Kicks (CTK) app. CTK is a free mobile app providing a virtual platform for pregnant people to conduct their daily kick counting. The study sample includes 1,147 pregnant people who used CTK during their third trimester and answered their end of pregnancy survey. Descriptive analyses, bivariate analyses, and logistic regression were used. Descriptive analyses were used to examine the number of kick counts using CTK and pregnant women's experiences with the app, their pregnancy, and childbirth. Bivariate analyses were used to examine the relationships between the frequency of kick counts and pregnant people's experience with their pregnancy and birth. Logistic regressions were used to model women's pregnancy experiences, including anxiety level related to their pregnancy and bonding with their baby.

**Results**

The study found that there is inadequate compliance with daily fetal movement counting recommendations in the third trimester among pregnant people. However, results showed that frequent use of fetal movement counting is associated with lower anxiety level related to their pregnancy, and more bonding with their baby. These positive pregnancy experiences are associated with healthy birth.

**Discussion**

To benefit from the impacts of fetal movement counting on positive pregnancy and birth experience, the app developers, public health agencies, and providers should implement strategies to encourage daily use of fetal movement counting.

## P12: Analysis of time series data and how stillbirth prevention efforts can help improve outcomes for moms

### Megan Aucutt

#### Healthy Birth Day, Inc., Des Moines, Iowa, USA

*BMC Proceedings 2023,***17(14):**P12

**Background**

There is little research on stillbirth prevention and little on whether there are effective methods in America for paying attention to a baby's movements in the third trimester. This study examines explicitly Count the Kicks, a stillbirth prevention program based in Iowa. The overall aim of the study was to assess whether there has been any discernible effect of Count the Kicks' activity on fetal deaths.

**Methods**

Researchers compiled datasets using the CDC Wonder Natality database. We extracted the overall monthly numbers of live births and fetal deaths in each month from 2005-2018 inclusive for Iowa and neighboring states and accessed aggregated data on confounding variables for the time periods in question. We used a time series approach, in which we evaluated trends in fetal deaths in relation to critical stages of the Count the Kicks campaign. The period was split into four tranches (date ranges are inclusive): 2005-2007 (before launching of CTK); 2008-2013 (early CTK, before app launch); 2014-2016 (following launch of the app); 2017-2018 (national media appearances resulting in a significant increase in app downloads).

**Results**

Only Iowa displayed an apparent decrease in stillbirths, which was statistically significant (OR 95% CI: 0.99 (0.99 to 1.00), p< 0.001. Stillbirth rates in Iowa are behaving differently from other neighboring states. In the US context, this alone merits further exploration. Additional studies on this stillbirth prevention program, including a study on hundreds of mHealth app users, show additional benefits such as increased bonding, reduced anxiety, and behavior change. We will present the findings of other smaller studies conducted on this program.

**Conclusions**

When we look at this study in the context of the American stillbirth rate --- we have one of the highest stillbirth rates in high-income countries. To demonstrate that we have a rate going down at 1 percent every three months in Iowa – which is much faster than the US population average – that tells us that something is happening in Iowa that is not happening in the rest of the United States. This makes a case for further research on a national scale. The study is critical --- and so is the real-life impact of stillbirth prevention efforts witnessed by doctors, nurses, and families. Our presentation will include case studies from expectant parents who used the stillbirth prevention program to alert their doctor to an emerging health issue.

